# Growth performance, carcass and meat quality of lambs supplemented different vegetable oils

**DOI:** 10.5713/ajas.18.0482

**Published:** 2018-11-27

**Authors:** Renata Miltko, Małgorzata Paulina Majewska, Grzegorz Bełżecki, Katarzyna Kula, Barbara Kowalik

**Affiliations:** 1 The Kielanowski Institute of Animal Physiology and Nutrition, Polish Academy of Sciences, Jabłonna 05-110, Poland

**Keywords:** Lamb Meat, Rapeseed Oil, Linseed Oil, Fatty Acids

## Abstract

**Objective:**

The purpose of this study was to evaluate the effect of rapeseed and linseed oil supplementations on performance and meat quality of lambs.

**Methods:**

The experiment was conducted on 18 growing (100-day-old) lambs of 19.7±1.9 kg live weight, assigned to 3 groups of 6 animals each. Control lambs were fed meadow hay and concentrate alone. Experimental animals additionally received rapeseed or linseed oils at a dose of 50 g/d. The lambs were slaughtered at an average body weight of 35.7±0.5 kg.

**Results:**

The dressing percentage was higher in lambs fed rapeseed oil. Total saturated fatty acids (SFA) and C15:0, C16:0, C17:0, C21:0, C24:0 were lower in *longissimus dorsi* muscle (MLD) in lambs fed linseed oil. Supplementation of diet with linseed oil decreased concentrations of total monounsaturated fatty acids and C16:1, C17:1, C18:1 *cis-9* in MLD. The concentrations of *n-3* polyunsaturated fatty acids (PUFA) and C18:3 *n-3*, C20:5 *n-3* in MLD were higher in lambs fed linseed oil than in other groups. Oils supplementation to diets resulted in increased concentration of C22:6 *n-3* in MLD. The inclusion of linseed oil into the diet increased the contents of total PUFA, *n-3* PUFA and C18:3 *n-3*, C20:5 *n-3*, C22:6 *n-3* in *semitendinosus* muscle in comparison to control. A tendency towards a lower n:6/n:3 ratio in MLD was observed when lambs were supplemented linseed oil.

**Conclusion:**

The supplementation of linseed oil to diets seems to reduce the concentration of SFA and increase the concentration of *n-3* PUFA. The *n-6*/*n-3* ratio is an important nutritional factor, and its value has been favorably decreased below 2, thereby achieving an important target related to human health. Due to these changes carcass fatty acid profile was improved, and so enhanced lamb meat healthy properties.

## INTRODUCTION

Ruminant meat is an important dietary component of human diets. Lamb and beef meat have been associated with an increased risk of cardiovascular diseases due to their high content of saturated fatty acid (SFA) [1]. The formation of large SFA is a result of the biohydrogenation process in the rumen during which bacteria convert unsaturated fatty acid (USFA) to SFA. So, the fatty acids (FA) occurring in the rumen are highly saturated and take part in the absorption as well as deposition of the fat in muscles. Nevertheless, such meat may be also a good dietary source of some nutrients with health benefits, including certain FA, particularly polyunsaturated fatty acids (PUFA≥C20) as well as conjugated linoleic acid (CLA) isomers. In addition, in a review by Chikwanha et al [2] it was shown that detrimental effects of rations rich in monounsaturated fatty acids (MUFA) are not known. In another study it was reported that oleic acid C18:1 *cis-9*, the primary *c*-MUFA found in lamb meat, was associated with beneficial effects related to cancer prevention as well as inflammatory and autoimmune diseases [3].

The decrease in SFA and increase of health-beneficial FA (PUFA and CLA) contents have been an important objective of ruminant meat studies. According to WHO/FAO [4], the ratio of PUFA and SFA is a significant indicator for nutritional evaluation of fat, with a recommendation of about 0.40. Beneficial effects of PUFA, such as eicosapentaenoic (EPA, C20:5 *n-3*) and docosahexaenoic (DHA, C22:6 *n-3*) acids, include anti-inflammatory, anti-atherogenic and anti-thrombotic actions [5]. It is essential to increase the consumption of EPA and DHA in human diets, because the synthesis of these FAs from dietary α-linolenic acid (ALA, C18:3 *n-3*) is very restricted.

In addition, lamb meat is among the richest natural sources of CLA. Many studies have shown that CLA demonstrate anti-carcinogenic, anti-diabetogenic and anti-atherogenic effects as well as other health-promoting properties [6]. Of twenty-eight known CLA isomers, cis9 trans-11 (rumenic acid) is the most common isomer exhibiting the highest biological activity. This isomer is formed during ruminal biohydrogenation of linoleic acid (LA, C18:2 *n-6*) to stearic acid (C18:0) and by endogenous conversion of vaccenic acid (VA, C18:1 trans-11) by Δ9 desaturase in tissues [7].

Some nutritional aspects, for example FA profile of the ra tion, lipid supplementation source, forage type as well as the forage/concentrate ratio in the diet are main driving factors of ruminant meat [2]. In ruminants some sources of FA such as ground oilseeds [8], whole oilseeds [9,10], extruded oilseeds [11] have already been studied. It was shown that the FA profile and concentration of CLA in meat may be affected by the form of the provided lipid. However, experiments evaluating kinds of oils are scarce [12,13] and the exact influence of particular form of fat on FA profile of lamb and beef meat is limited.

It was hypothesised that diet supplementation with rapeseed oil rich in oleic acid, or linseed oil, a good source of linolenic acid, can reduce the concentration of SFA and increase *n-3* PUFA.

So, the objective of this study was to compare the effect of rapeseed and linseed oils on lamb growth, carcass traits and FA composition of *longissimus dorsi* and *semitendinosus* muscles (MS) with implications to human health.

## MATERIALS AND METHODS

All procedures were approved by the Local Ethics Committee for Animal Experimentation in Warsaw (No 51/2009).

### Animals and feed

The study was conducted on 18 growing (100-day-old) male Corridale lambs of 19.7±1.9 kg live weight. The animals were assigned to 3 groups of 6 lambs each (according to initial body weight) in a randomized complete block design. The animals were housed in individual pens on wheat straw with separate facilities for feeding. Control animals were fed a basal diet containing meadow hay, concentrate – crushed barley and soybean oilmeal and vitamin-mineral mixture (Polfamix - OK, Trouw Nutrition, Poland); experimental lambs were fed a basal ration and 5% rapeseed or linseed oils. Oils were stored out of direct sunlight at 4°C and supplements were mixed into the concentrate. Dietary compositions are given in Table 1 and FA profiles of rapeseed and linseed oils are presented in Table 2. Daily rations were offered in two equal portions at 8:00 and 16:00. The lambs had free access to water and blocks of salt containing microelements. Since animals were provided with a limited amount of feed, no orts were observed.

The animals were weighed every two weeks during fatten ing in order to adjust diets to body weight. The lambs were weighed just before morning feeding. Feed intake, utilization as well as daily live weight gains were recorded. Daily feed intake was compared with the requirement for 250 g gain and adjusted to the average daily gain (ADG) obtained for each diet [14].

### Slaughter and carcass measurements

The lambs were slaughtered after about 55 days at an average body weight of 35.7±0.5 kg, in accordance with standard practises [15]. Before slaughter, animals were starved for 12 h, but had free access to drinking water. Afterwards, non-carcass components, gastrointestinal tracts and viscera were removed. Selected internal organs (liver, heart, and kidneys) were weighted. Carcasses were kept at 4°C for 24 h and a simple dissection of their right halves was carried out. Thereafter, carcasses were split into commercial cuts, i.e. loin, shoulder and leg; selected cuts were weighted. The samples of *longissimus dorsi* muscle (MLD) from the 10th to 13th rib and MS were taken to analyse the chemical composition and FA profile. Muscle samples were minced, packed into polyethylene bags and stored at −30°C until analysis.

### Chemical analysis

The chemical composition of feed and muscle samples was determined according to standard methods [16]. The samples were analysed for dry matter (DM) (AOAC official method 934.01), crude protein (CP, total nitrogen×6.25, AOAC official method 954.01), crude fat (AOAC official method 960.39), ash (AOAC official method 930.05). Starch concentration in feeds was determined according to the official method of AOAC 920.40; natural detergent fibre (NDF) and acid detergent fibre (ADF) were analysed according to van Soest et al [17].

Lipids from oils and muscles were extracted with chloroform-methanol (2:1), according to Folch et al [18]. After filtration through a filter paper, 800 μL of filtrate was transferred into glass vials. Thereafter, vials were evaporated to dryness under nitrogen stream in a heating block at 50°C. The samples were saponified with 1.5 mL of 0.5 M KOH in methanol in a heating block at 75°C. Next, the samples were esterified with 3.0 mL of 4% solution of SOCl_2_ in methanol. Methyl esters were extracted with 1.0 mL of n-heptane and salted out with NaCl to separate the organic layer. Subsequently, 300 μL methyl esters were transferred into glass vials and 600 μL of n-heptane was added. The vials were kept at −30°C until fatty acid methyl esters (FAME) analyses. The FAME were separated and quantified using a gas chromatograph (Shimadzu GC-2010, Kyoto, Japan) equipped with a flame-ionisation detector (FID), injection port and a BPX70 fused silica capillary column (120 m ×0.25 mm i.d.×0.25 μm film thickness [SHIM-POL, Izabelin, Poland]). Helium was used as the carrier gas, operated at a constant pressure of 223.4 kPa and flow rate of 1 mL/min. Injector and FID temperatures were maintained at 200°C and 240°C, respectively. FAME profile was analysed using the temperature gradient programme in a 1-μL sample at a split ratio of 10:1, as described by Czauderna et al [19]. The analysis started at 70°C for 4 min increasing at a rate of 12°C/min to 150°C, which was maintained for 6 min; then increased at 8°C/min to 168°C, maintained for 27 min and increased by 0.75°C/min to 190°C and maintained for 10 min; then increased again at a rate of 1.8°C to 210°C, maintained for 15 min; elevated at a rate of 6°C/min to 234°C, which was kept for 4 min and finally increased at a rate of 6°C/min to 236 and maintained for 20 min. FAME peaks were identified by comparison with Supelco 37 Component FAME Mix (Supelco Inc., Bellefonte, PA, USA) and CLA standard mix (Sigma-Aldrich, Inc., St. Louis MO, USA).

The Δ^9^-desaturase index was calculated as (C14:1 *cis-9*+C16:1 *cis-9*+C18:1 *cis-9*+C18:2 *cis-9*, *trans-11*)/(C10:0+C14:0+C14:1 *cis-9*+C16:0+C16:1 *cis-9*+C18:0+C18:1 *cis-9*+C18:1 *trans-11*+ C18:2 *cis-9*, *trans-11*), according to Brogna et al [20].

### Statistical analysis

Data were expressed as means and pooled standard error of the mean. The normality of the data and homogeneity of variance were tested using the Shapiro-Wilk and Brown-Forsythe tests, respectively. Data were analyzed using one-way analysis of variance in the general linear model of Statistica (Statistica 10.0 software, StatSoft, Kraków, Poland) with treatment (diet) as a fixed effect.

The effects were considered to be significant at p≤0.05 or p<0.01 and p values<0.10 were considered trends in the data. The individual means were compared by the Tukey HSD post-hoc test (Statistica 10.0 software, StatSoft, Poland).

## RESULTS

### Animal performance

Nutrient composition of the diets is given in Table 1. As expected, crude fat content and feed unit of maintenance and meat production were higher for oil-supplemented than control ration. Furthermore, the contents of CP, crude ash and starch as well as NDF and ADF (as g/kg DM) were lower for oil-supplemented than control diet. Daily DM intake was higher when oils were supplemented in comparison to the control ration (1.05 vs 1.00 kg DM/d).

Lambs in different oil supplementation treatments were similar in terms of ADG (p = 0.816) and feed conversion ratio (p = 0.982) (Table 3). Significantly higher (p = 0.039) dressing percentage (DP) and a tendency towards increased hot carcass and right half carcass weights (p = 0.100 and p = 0.060, respectively) were recorded in lambs fed the rapeseed oil diet. The weights of carcass components, i.e. loin (p = 0.718), shoulder (p = 0.472), and leg (p = 0.378), did not change significantly when oils were supplemented.

Chemical composition of muscles was not affected by oil supplementation (Table 4); however, a tendency towards increased CP in MS (p = 0.097) was observed when lambs were fed rapeseed oil in comparison with linseed oil. The weights of internal organs (liver [p = 0.674], heart [p = 0.726], and kidneys [p>0.100]) did not differ between the three treatments.

### Oil supplementation effects on saturated fatty acids

The concentrations of C15:0 (p = 0.003), C16:0 (p = 0.045), C17:0 (p = 0.027), C21:0 (p<0.001), C24:0 (p<0.001), and as a consequence, of total SFA (p = 0.049) were significantly lower in MLD in the group receiving linseed oil than in the control group (Table 5). When linseed oil was added to the diet, C24:0 concentration in MS was significantly lower (p = 0.006) as compared to the control ration (Table 6). Total SFA in MS was not influenced by oil supplementations (p = 0.975).

### Oil supplementation effects on monounsaturated fatty acids

Linseed oil supplementation significantly decreased the concentration of C16:1 (p = 0.024), C17:1 (p<0.001), C18:1 *cis-9* (p = 0.006) and total MUFA (p = 0.011) in MLD compared to rapeseed oil and control groups (Table 5). The addition of rapeseed oil to the diets significantly decreased C15:1 concentration (p = 0.006) in MLD in comparison to control and linseed oil rations. Moreover, the addition of linseed oil to lamb diets significantly increased C24:1 contents (p = 0.003) in MS than in control and did not influence other MUFA (p>0.100) (Table 6).

### Oil supplementation effects on polyunsaturated fatty acids

The concentrations of ALA (p<0.001), EPA (p = 0.010) and total *n-3* PUFA (p = 0.011) in MLD were significantly higher when linseed oil was provided in comparison to control and lambs fed rapeseed oil (Table 5). In turn, the content of C20:3 in MLD was significantly lower (p = 0.001) in animals fed linseed oil than in control ones. Oil supplementations to animal diets caused significantly increased concentration of DHA (p = 0.018) in MLD when compared to the control diet. The addition of linseed oil to the diets significantly increased concentrations of ALA (p<0.001), EPA (p = 0.007) as well as total *n-3* PUFA (p = 0.001) and sum of PUFA (p = 0.004) in MS compared to control and rapeseed oil groups (Table 6). Further, the addition of oils to the rations significantly increased DHA concentration in MS (p = 0.008).

### Oil supplementation effects on n:6/n:3 and PUFA/SFA ratios and Δ^9^-desaturase index

A tendency towards a lower n:6/n:3 ratio (p = 0.054) in MLD was observed when lambs were fed linseed oil in comparison to control and rapeseed oil diets (Table 5). The addition of linseed oil to the diets significantly increased PUFA/SFA ratio (p = 0.002) in MLD as compared to control and rapeseed oil groups (Table 5). The Δ^9^-desaturase index was the lowest in MLD (p = 0.062) and MS (p = 0.013) when lambs were fed linseed oil (Tables 5, 6).

## DISCUSSION

### Animal performance

Generally, the type of dietary fat supplementation has no effect on growth performance and carcass traits of lambs or goats [21,22,10]. However, in the present study, rapeseed oil supplementation was associated with a higher DP, carcass and half carcass weights. These results can be explained by the high energy level, crude fat concentration in lamb diets and higher DM intake. These observations are consistent with the results of Peng et al [9], who reported beneficial effects of rapeseed oil supplementation to diet on DP and carcass weight.

No differences were identified between control and treatment groups in terms of muscle (MLD, MS) chemical composition. Similar results were reported in relation to the effect of supplementation of different forms of sunflower products on DM, CP, fat and ash contents in muscles [10].

### Meat fatty acid composition

The content and profile of specific FA in meat are important factors in assessing its nutritional quality. The FA profiles of adipose tissue and muscles are determined by *de novo* lipogenesis, desaturation as well as ration lipid composition, feeding duration and difference in the utilization of various FA by the animal body [8,11].

#### Saturated fatty acids

Linseed oil supplementation reduced SFA (C15:0, C16:0, C17:0, C21:0, C24:0) in MLD, but decreased only C24:0 concentration in MS. Linseed oil supplementation lowers SFA levels, especially C15:0, C16:0, and C17:0, which is desirable, because this FA is the major hypercholesterolemic FA and it is associated with a higher risk of cardiovascular diseases and type 2 diabetes [2]. According to Yang et al [23], decreased SFA in muscles of ruminants fed linseed oil in the ration was presumably caused by *de novo* synthesis inhibition by a higher percentage of exogenous FA in the metabolic pool. Additionally, SFA reduction in muscles derived from lambs fed linseed oil may also reflect an incomplete biohydrogenation process, as an effect of the high consumption of unprotected fats rich in linolenic acid. Czauderna et al [24] reported a reduction in the concentration of SFA in lamb *longissimus dorsi* and *biceps femoris* muscles after supplementing control diet with linseed oil. These authors suggested that the oil stimulated peroxidation damage and/or catabolism of FA. Our results are consistent with those of Gallardo et al [22], who observed a greater decrease in C15:0, C16:0, C17:0, and C24:0 concentrations in intramuscular fat of lambs sucking ewes and receiving diets supplemented with linseed oil than in the control animals. On the other hand, Ebrahimi et al [13] did not find significant changes in SFA concentration in the subcutaneous adipose tissue of goats fed diet with different levels of linseed oil.

#### Monounsaturated fatty acids

MUFA contain a single double bond in the *cis-* or *trans*-configuration. Linseed oil inclusion into diet causes changes in the MUFA profile in MLD, in particular reduces oleic acid (C18:1 *cis-9*), which is the main MUFA. Bessa et al [12] also found that linseed oil in diets decreased C18:1 *cis-9* concentration in *longissimus thoracis* in comparison to un-supplemented diets. Oleic acid reduction in ruminant tissues is often associated with PUFA supplementation in the ration [25]. Such situation can be due to either the absence of C18:0 (stearic acid) desaturated by Δ^9^-desaturase or the inhibition of enzyme activity by high PUFA contents [26]. In our experiment, the quantity of stearic acid in the ration was low, thus its origin here was primarily derived from endogenous *de novo* synthesis, and as an end-product of C18 PUFA biohydrogenation in the rumen [12]. In PUFA-supplemented ruminant diets, *de novo* FA synthesis is reduced; PUFA biohydrogenation becomes incomplete, and biohydrogenation intermediates start to accumulate, causing lower C18:0 yield, as shown in the current study. Moreover, Mosley et al [27] documented in the *in vitro* study a direct octadeceonoate *cis-trans* isomerization in mixed rumen bacteria, capable of converting oleic acid into *trans-11* as well as other *trans* isomers. In our study, insignificant increase of C18:1 *trans 11* in MLD and MS was observed when lambs were fed linseed oil in comparison to other diet. However, C18:0 reduction in MLD of lambs fed linseed oil was small. In contrast to our findings, Gallardo et al [22] did not find any significant changes in C18:1 *cis-9* concentrations of intramuscular fat of lamb sucking ewes fed diets supplemented with linseed oil.

#### Polyunsaturated fatty acids

Lipids in vegetable oils are characterized by a high content of USFAs. Feeding vegetable oils, such as linseed, rapeseed or fish oils decreases SFA concentration in meat and increases the content of valuable PUFA especially ALA, EPA, DHA. Feeding strategies that induce a decrease in saturated lipids and an increase in *n-3* FA of intramuscular fat would improve the value of lamb meat. Conversion capacity of C18:3 *n-3* to health-promoting long chain *n-3* PUFA is limited in humans [2], which reinforce the significance of its dietary supply. The increase in the concentration of long chain *n-3* PUFA coincided with increase in dietary C18:3 *n-3* concentration of linseed oil rations. This pattern was consistent with other trials where lambs were fed oils rich in C18:3 *n-3* [28,13]. Lambs fed diet containing linseed oil accumulated a significantly higher amount of total *n-3* PUFA in their muscles than lambs fed other diets. Long chain *n-3* PUFA in muscles are metabolic products of C18:3 *n-3*. Competition between C18:3 *n-3* and C18:2 *n-6* for desaturation and elongation enzymes may affect the conversion to long chain derivatives, while the high concentration of *n-3* PUFA is likely due to the preference of these enzymes for C18:3 *n-3*. On the other hand, Bessa et al [12] and Gallardo et al [22] showed that diet supplementation with linseed oil, high in C18:3 *n-3*, was coupled with decreased amounts of long chain *n-3* PUFA in *longissimus thoracis* and intramuscular fat, suggesting an inhibition of C18:3 *n-3* metabolism. Bessa et al [12] explained that this inhibition could have been caused by the repression of gene expression mediated by linolenic acid.

In our experiment, there was a decrease in the amounts of C20:3 *n-6* in MLD muscle in lambs fed linseed oil. According to Nuernberg et al [29], this could be explained by the lower total FA concentration in the grass-based diet, allowing long chain phospholipid FA to dominate total lipids. Hence, it cannot be precluded that in our study the amount of total FA in meadow hay varied, all the more that lamb fattening lasted more than 50 days.

#### Nutritional quality of meat

The *n-6*/*n-3* and PUFA/SFA ratios are used to evaluate the nutritional value of fat for human consumption. The n:6/n:3 ratio is strongly dependent on the FA profile of the ration fed to ruminants. This ratio is particularly beneficial in the meat of ruminants that consume forages or oilseed with an increased C18:3 content [2]. Lowering the *n-6*/n:3 coefficient in food production has been recommended to prevent or modulate certain human diseases, and it should range between 1 and 4 [4]. Our results were within this range, but the n:6/n:3 ratio found in MLD and MS was the lowest when lambs were fed linseed oil. These results are consistent with findings of Jerónimo et al [28], who reported that the n:6/n:3 ratio in MLD of lambs was significantly decreased when dietary sunflower oil was replaced with linseed oil. In the group with linseed oil, 59.55% of total *n-3* PUFA were long chain *n-3* PUFA (C20:3, C20:5, and C22:6). Ebrahimi et al [13] believed that this could be important considering that health benefits of *n-3* PUFA are predominantly associated with long chain *n-3* PUFA, as C18:3 *n-3* metabolism is restricted in humans.

Usually lamb meat has a high SFA content and low PUFA/SFA values. Increasing the PUFA concentration in the ration, by including a source rich in either *n-6* or *n-3* PUFA generally improves the PUFA/SFA ratio. This was also observed in the present study, and in oil diets, the PUFA/SFA ratio was lower than 0.45, so as recommended by the WHO/FAO [4].

## CONCLUSION

According to the results of the present study, FA profile in muscles was depended on oils supplemented to lamb diets. Additionally, the supplementation of linseed oil to diets seems to reduce the concentration of SFA and increase the concentration of *n-3* PUFA, particularly ALA, EPA, and DHA in meat. The *n-6*/*n-3* ratio is an important nutritional factor, and its value has been favorably decreased below 2, thereby achieving an important target related to human health. Due to these changes carcass FA profile was improved, and so enhanced lamb meat healthy properties.

## Figures and Tables

**Table 1:** Composition of diets

Items	Diet	

	Control	Oil1)
Ingredients (g/kg DM)		
Meadow hay	369	350
Crushed barley	452	431
Soybean oilmeal	159	152
Oil	-	48
Vitamin-mineral premix2)	20	19
Chemical composition (g/kg DM)		
DM (g/kg)	874	899
Crude protein	175	164
Crude fat	22.9	69
Starch	342	324
Crude ash	45.4	43.1
NDF	442	421
ADF	191	182
UFV (kg)	1.0	1.1

DM, dry matter; NDF, neutral detergent fibre; ADF, acid detergent fibre; UFV, feed unit of maintenance and meat production.

1) Rapeseed or linseed oils.

2) Contained per kg: vit A 300,000 IU; vit D_3_ 30,000 IU; vit E 1.5 g; Mn 3 g; Zn 2.5 g; Co 0.015 g; Se 0.003 g; Ca 240 g; Na 60 g; P 120 g; Mg 65 g.

**Table 2:** Contents of some fatty acids in rapeseed and linseed oils (g FAME/100 g oil)

Fatty acid	Rapeseed oil	Linseed oil
C16:0 palmitic	1.9	1.9
C18:0 stearic	2.3	1.6
C18:1 *n-9* oleic	63.7	28.5
C18:2 *n-6* linoleic	23.0	17.5
C18:3 *n-3* linolenic	8.8	50.1

FAME, fatty acid methyl esters.

**Table 3:** Animal performance, carcass characteristic and carcass components of major cuts of lambs

Item	Control	Rapeseed oil	Linseed oil	SEM	p-value
Daily feed intake (kg/d)	1.15	1.2	1.2	-	-
Initial live weight (kg)	19.9	19.7	19.5	0.46	0.941
Slaughter weight (kg)	35.7	35.7	35.7	0.12	0.955
Days of fattening	56.3	54.3	55.5	-	-
ADG (g/d)	280	295	293	8.7	0.816
FCR	4.1	4.1	4.1	0.13	0.982
Hot carcass weight (kg)	14.3	15.1	14.7	0.16	0.100
Right half of carcass (kg)	6.4	7.1	6.9	0.12	0.060
DP (%)	40.0^a^	42.4^b^	41.2^ab^	0.40	0.039
Loin (kg)	0.3	0.4	0.3	0.01	0.718
Shoulder (kg)	1.2	1.1	1.1	0.02	0.472
Leg (kg)	1.7	1.8	1.7	0.05	0.378

SEM, standard error of mean; ADG, average daily gain; FCR, feed conversion ratio; DP, dressing percentage (hot carcass weight×100/slaughter weight).

ab Means in a row with different superscripts are significantly different (p<0.05).

**Table 4:** Chemical composition of muscles (g/100 g) and internal organs weight (g)

Item	Control	Rapeseed oil	Linseed oil	SEM	p-value
MLD					
Dry matter	23.9	23.9	23.9	0.14	0.994
Crude protein	19.3	19.1	19.1	0.14	0.818
Crude ash	1.1	1.1	1.1	0.01	0.317
Crude fat	2.4	2.4	2.7	0.10	0.468
MS					
Dry matter	24.0	24.5	23.1	0.17	0.471
Crude protein	19.4	19.7	18.9	0.14	0.097
Crude ash	1.1	1.1	1.1	0.01	0.121
Crude fat	1.9	2.6	2.4	0.16	0.286
Internal organs					
Liver	618	611	642	14.1	0.674
Heart	160	165	168	4.0	0.726
Right kidney	46.0	50.0	46.2	2.04	0.623
Left kidney	47.3	47.3	46.5	1.57	0.972

SEM, standard error of mean; MLD, *longissimus dorsi* muscle; MS, *semitendinosus* muscle.

**Table 5:** The fatty acid composition of *longissimus dorsi* muscle in lambs (mg FAME/100 g muscle)

Fatty acid	Control	Rapeseed oil	Linseed oil	SEM	p-value
SFA					
C10:0	nd	nd	nd	-	-
C14:0	50.1	43.0	33.7	3.69	0.196
C15:0	21.9^a^	19.7^ab^	17.0^b^	0.68	0.003
C16:0	514^a^	449^ab^	388^b^	21.6	0.045
C17:0	63.2^a^	48.6^ab^	45.8^b^	3.04	0.027
C18:0	473	411	382	20.5	0.188
C20:0	6.6	5.3	6.4	0.50	0.565
C21:0	9.0^a^	7.4^a^	4.5^b^	0.55	<0.001
C24:0	4.2^a^	3.0^ab^	2.6^b^	0.20	<0.001
∑SFA1)	1,142^a^	987^ab^	880^b^	45.6	0.049
MUFA					
C14:1 *cis-9*	8.9	7.3	5.9	0.56	0.078
C15:1 *cis10*	3.7^a^	1.9^b^	2.1^a^	0.28	0.006
C16:1 *cis-9*	27.4^a^	25.7^a^	18.0^b^	1.62	0.024
C17:1 *cis-10*	13.3^a^	9.8^b^	7.0c	0.73	<0.001
C18:1 *trans-11* (VA)	23.4	20.5	30.6	2.55	0.251
C18:1 *cis-9*	792a	766^a^	582^b^	33.00	0.006
C24:1 *cis-15*	8.3	8.8	10.1	0.32	0.053
∑MUFA2)	877^a^	840^a^	656^b^	35.4	0.011
PUFA					
C18:2 *n-6 trans-9 trans-12*	6.4	6.1	11.5	1.02	0.118
C18:2 *n-6 cis-9 cis-12* (LA)	110	101	108	2.9	0.449
C18:2 *cis-9 trans-11* (CLA)	8.6	7.0	8.4	0.66	0.565
C18:3 *n-3* (ALA)	10.2^a^	11.1^a^	28.4^b^	2.33	<0.001
C20:3 (*n-6*)	3.4^a^	2.9^b^	2.3^c^	0.12	<0.001
C20:3 (*n-3*)	44.4^a^	38.0^ab^	32.0^b^	1.64	0.001
C20:5 (*n-3* EPA)	3.4^a^	4.6^ab^	6.4^b^	0.46	0.010
C22:6 (*n-3* DHA)	2.2^a^	3.3^b^	3.4^b^	0.21	0.018
∑PUFA3)	180	167	192	4.6	0.093
∑PUFA *n-6*4)	120	110	122	3.1	0.269
∑PUFA *n-3*5)	60.2^a^	57.0^a^	70.2^b^	1.98	0.011
*n-6*/*n-3*	2.0	1.9	1.7	0.06	0.054
PUFA/SFA	0.16^a^	0.17^a^	0.21^b^	0.009	0.002
Δ^9^-desaturase index	0.44	0.47	0.42	0.007	0.062

FAME, fatty acid methyl esters; SEM, standard error of mean; SFA, saturated fatty acids; nd, not detectable; MUFA, monounsaturated fatty acids; VA, vaccenic acid; PUFA, polyunsaturated fatty acids; LA, linoleic acid; CLA, conjugated linoleic acid; ALA, linolenic acid; EPA, eicosapentaenoic acid; DHA, docosahexaenoic acid;

1) Sum of SFA: C14:0; C15:0; C16:0; C17:0; C18:0; C20:0; C21:0; C24:0.

2) Sum of MUFA: C14:1 *cis-9*; C15:1 *cis-10*; C16:1 *cis-9*; C17:1 *cis-10*; C18:1 (VA); C18:1 *cis-9*; C24:1 *cis-15*.

3) Sum of PUFA: C18:2 *trans-9 trans-12*; C18:2 (LA); C18:3 (ALA); C20:3 *n-6*; C20:3 *n-3*; C20:5 (EPA); C22:6 (DHA).

4) Sum of PUFA *n-6*: C18:2 *trans-9 trans-12*; C18:2 (LA); C20:3 *n-6*.

5) Sum of PUFA *n-3*: C18:3 (ALA); C20:3 *n-3*; C20:5 (EPA); C22:6 (DHA).

abc Means in a row with different superscripts are significantly different (p<0.05).

**Table 6:** The fatty acid composition of *semitendinosus* muscle in lambs (mg FAME/100 g muscle)

Fatty acid	Control	Rapeseed oil	Linseed oil	SEM	p-value
SFA					
C10:0	nd	nd	nd	-	-
C14:0	36.4	41.1	36.3	2.89	0.774
C15:0	25.2	24.0	23.9	0.59	0.694
C16:0	384	383	364	24.7	0.941
C17:0	45.2	37.7	39.8	3.08	0.641
C18:0	324	328	328	20.7	0.996
C20:0	4.9	5.2	5.4	0.51	0.906
C21:0	8.9	8.2	5.7	0.66	0.089
C24:0	4.4^a^	3.8^ab^	2.9^b^	0.22	0.006
∑SFA1)	833	831	806	50.8	0.975
MUFA					
C14:1 *cis-9*	6.4	6.6	6.0	0.39	0.816
C15:1 *cis10*	2.6	2.1	1.7	0.28	0.474
C16:1 *cis-9*	21.6	23.7	18.2	1.49	0.346
C17:1 *cis-10*	10.1	8.1	6.6	0.74	0.205
C18:1 *trans-11* (VA)	16.8	20.1	25.0	2.53	0.442
C18:1 *cis-9*	614	639	542	40.8	0.648
C24:1 *cis-15*	8.5^a^	10.4^ab^	12.5^b^	0.56	0.003
∑MUFA2)	680	710	612	44.3	0.697
PUFA					
C18:2 *n-6 trans-9 trans-12*	5.4	5.6	10.4	0.98	0.055
C18:2 *n-6 cis-9 cis-12* (LA)	114	122	135	3.8	0.053
C18:2 *cis-9 trans-11* (CLA)	6.3	6.9	7.1	0.68	0.906
C18:3 *n-3* (ALA)	9.4^a^	12.5^a^	29.8^b^	2.79	<0.001
C20:3 (*n-6*)	3.8	3.4	3.4	0.12	0.296
C20:3 (*n-3*)	52.0	50.2	46.4	1.49	0.286
C20:5 (*n-3* EPA)	3.6^a^	5.1^ab^	8.4^b^	0.74	0.007
C22:6 (*n-3* DHA)	2.8^a^	4.2^b^	4.6^b^	0.29	0.008
∑PUFA3)	191^a^	203^a^	238^b^	7.1	0.004
∑PUFA *n-6*4)	123	131	149	4.3	0.121
∑PUFA *n-3*5)	67.8^a^	72.2^a^	89.2^b^	3.11	0.001
*n-6/n-3*	1.8	1.8	1.7	0.04	0.205
PUFA/SFA	0.23	0.24	0.29	0.016	0.227
Δ^9^-desaturase index	0.46^a^	0.47^a^	0.43^b^	0.006	0.013

FAME, fatty acid methyl esters; SEM, standard error of mean; SFA, saturated fatty acids; nd, not detectable; MUFA, monounsaturated fatty acids; VA, vaccenic acid; PUFA, polyunsaturated fatty acids; LA, linoleic acid; CLA, conjugated linoleic acid; ALA, linolenic acid; EPA, eicosapentaenoic acid; DHA, docosahexaenoic acid.

1) Sum of SFA: C14:0; C15:0; C16:0; C17:0; C18:0; C20:0; C21:0; C24:0.

2) Sum of MUFA: C14:1 *cis-9*; C15:1 *cis-10*; C16:1 *cis-9*; C17:1 *cis-10*; C18:1 (VA); C18:1 *cis-9*; C24:1 *cis-15*.

3) Sum of PUFA: C18:2 *trans-9 trans-12*; C18:2 (LA); C18:3 (ALA); C20:3 *n-6*; C20:3 *n-3*; C20:5 (EPA); C22:6 (DHA).

4) Sum of PUFA *n-6*: C18:2 *trans-9 trans-12*; C18:2 (LA); C20:3 *n-6*.

5) Sum of PUFA *n-3*: C18:3 (ALA); C20:3 *n-3*; C20:5 (EPA); C22:6 (DHA).

ab Means in a row with different superscripts are significantly different (p<0.05).
